# How far are we from bringing intensive care bundle for intracerebral hemorrhage into the real-world setting? A 5-year population based-study

**DOI:** 10.1007/s10072-025-08113-x

**Published:** 2025-03-31

**Authors:** Paola Colantuono, Lucio D’Anna, Matteo Foschi, Michela Adipietro, Stefania Lancia, Leondino Mammarella, Simona Sacco, Raffaele Ornello

**Affiliations:** 1 Department of Biotechnological and Applied Clinical Sciences, University of L’Aquila, Via Vetoio 1, L’Aquila, Italy; 2 Department of Stroke and Neuroscience, Charing Cross Hospital, Imperial College London NHS Healthcare Trust, London, United Kingdom; 3 Department of Brain Sciences, Imperial College London, London, United Kingdom; 4 Servizio Flussi Informativi e Statistica Sanitaria, Azienda Sanitaria Locale Avezzano-Sulmona- L’Aquila, L’Aquila, Italy

**Keywords:** Intracerebral hemorrhage, Bundle of care, Systolic blood pressure, Prognosis

## Abstract

**Introduction:**

Comprehensive care bundles including rapid blood pressure management, anticoagulation reversal, neurosurgical consultation, control of blood glucose and body temperature, can improve short- and medium-term outcomes in patients with intracerebral hemorrhage (ICH). This study assessed how the acute management of ICH practices evolved in a real-world setting over five years characterized by global changes in ICH care.

**Methods:**

This study analysed ICH cases from a population-based stroke registry between 2018 and 2022. We collected demographic and clinical data, focusing on key parameters of ICH management, such as systolic blood pressure, anticoagulation reversal, neurosurgical referrals, blood glucose, and body temperature. We also examined yearly trends in control of parameters over time.

**Results:**

We included 460 patients with ICH (55.4% male, median age 79 years, interquartile range 69–85). At onset, 266 patients (57.8%) had high SBP (SBP ≥ 140 mmHg), 286 (70.3%) hyperglycemia (blood glucose ≥ 108 mg/dL), and 63 (17.3%) hyperpyrexia (body temperature ≥ 37.0*C). Anticoagulation was reversed in 21.4% of anticoagulated patients within 24 h. Neurosurgical referrals were made for 84.6% of patients while only 12.4% underwent surgery. From 2018 to 2022, anticoagulation reversal rates increased from 0 to 88.9% (*p* < 0.001), while neurosurgical referrals not followed by surgery decreased from 79.5 to 55.7% (*p* < 0.001).

**Conclusions:**

This real-world study demonstrates suboptimal management of key factors associated with ICH prognosis; nevertheless, it highlights improvement over time. There is a need for structured interventions to improve the timely and consistent application of simple yet effective measures yielding the potential to improve patients’ outcomes.

**Supplementary Information:**

The online version contains supplementary material available at 10.1007/s10072-025-08113-x.

## Introduction

Intracerebral hemorrhage (ICH) is a leading cause of morbidity and mortality worldwide, with an estimated 11.9 million incident cases, accounting for 26% of all strokes [1]. Unlike ischemic stroke, there are no specific, widely adopted acute therapies for ICH. Clinical studies demonstrated that a “bundle of care” approach, including a series of urgent and simple management strategies performed collectively, can significantly improve ICH prognosis [2, 3].

In 2019 it was shown that the implementation of a bundle of care reduced ICH-related disability or death within 30 days in an English cohort [4]. Key components of that bundle of care included rapid blood pressure control, prompt reversal of anticoagulation in anticoagulated patients and neurosurgical referral for selected patients [5, 6]. Thereafter, the Intensive Blood Pressure Reduction in Acute Cerebral Hemorrhage Trial 3 (INTERACT3) confirmed that the early application of a bundle of care approach within the first few hours of symptom onset can have a substantially positive impact on 6-month prognosis of patients with ICH [7]. Notably, the INTERACT3 trial added the implementation of hyperglycemia and hyperpyrexia management to the bundle of care. 

In this study we aimed to evaluate the management of key components of the bundle of care approach over a 5-year period where this new approach to acute ICH management was developed.

## Methods

### Study design and population

We followed the Strengthening the Reporting of Observational Studies in Epidemiology (STROBE) to report the results of this study [8]. Our study is part of a prospective population-based stroke registry performed in the district of L’Aquila (298,343 inhabitants) [9, 10], in central Italy. The district of L’Aquila is a mountainous area served by four public hospitals with 24/7 availability of brain computed tomography (CT); two hospitals have neurology wards and one a neurosurgical ward. Medical care in the district is completely free of charge with easy access to medical services for the acute phase of a stroke. The registry complies with epidemiological criteria for stroke incidence studies and was approved by the Internal Review Board of the University of L’Aquila with protocol numbers 13/2018 and 57/2019. The registry includes all cases of cerebrovascular events occurring in the district, which are regularly reported by local physicians, validated by the study staff, and followed-up.

Patients were treated according to the routine clinical practice and following national and international guidelines. Among all cases of stroke recorded in the Registry since 2011, we included all cases of ICH occurring in the district of L’Aquila over a 5-year period, from 1 January 2018 until 31 December 2022. Diagnoses of ICH were validated according to the presence of focal neurological deficits along with concomitant evidence of intraparenchymal hemorrhage at brain imaging. We included patients with either first-ever or recurrent ICH and excluded those with hemorrhagic transformation of a cerebral infarction. Patients with primary subdural/epidural hematoma or traumatic ICH or hemorrhage due to a tumor were also excluded.

### Case-finding procedures

We performed active monitoring of inpatient and outpatient health services to identify each ICH event. Furthermore, all ICH cases were identified by a senior physician within 7 days of symptoms onset and then confirmed by a consulting neurologist. Admission and discharge lists, as well as emergency services, neuroradiology and neurophysiology services were checked. We also reviewed the records of patients with other possible differential diagnoses, such as transient ischemic attack (TIA), dizziness, vertigo, confusion, seizures, headache, and transient global amnesia. Nearby hospitals, rehabilitation, and long-term care services were also monitored. Death certificates were examined monthly and clinical data of all patients who died with an ICH diagnosis, where not otherwise included in the registry, were incorporated. Hot (active identification of all events at the time they occurred) and cold (retrospective identification) pursuits were combined in the ascertainment of cases to ensure a complete identification.

### Data collection and follow-up

Demographic and clinical data were collected through systematic consultation of medical records and stored in a computerized database in an anonymized form using Research Electronic Data Capture (REDCap) [11]. We collected medical history, cardiovascular, and neurological evaluations. Furthermore, we recorded level of consciousness at ICH onset according to the Glasgow Coma Scale (GCS) score and clinical severity on admission based on the National Institutes of Health Stroke Scale (NIHSS) score. For the purpose of the present study, we collected systolic blood pressure (SBP) values, blood glucose levels, and body temperature at ICH onset and at 24 h from symptom onset. Values at onset were first measured in the ambulance or in the Emergency Room, while values at 24 h were collected within the wards in those who were hospitalized. We considered SBP as *high* if being ≥ 140 mmHg and *optimal* if being < 140 mmHg. We considered blood glucose values as *hyperglycemia* if being ≥ 6.0 mmol/L (≥ 108 mg/dL) [12]. We considered body temperature as *hyperpyrexia* if being ≥ 37.0 °C. Together with those data, we collected data on administration of anticoagulation reversal among people taking anticoagulant therapy within 24 h from onset. We also recorded neurosurgical referrals and indications for surgery (intraventricular drainage or ICH evacuation). Thirty-day case-fatality was also assessed. All those parameters were recorded in the overall population and for each year of observation to assess time trends.

### Statistical analysis

Descriptive statistics were reported as absolute numbers with percentages or medians and interquartile ranges (IQRs), as appropriate. Comparisons between values at ICH onset and those at 24 h were performed via the chi-squared test. As we assumed that SBP, glycemia, and temperature were not measured in all patients, we performed comparisons to test whether the characteristics and outcomes differed in patients with and without those parameters measured at baseline. Time trends were assessed using chi-square test for trends. The thirty-day case-fatality rates were reported as number and percentages with the corresponding 95% CIs. Thirty-day survival after ICH according to key parameters of ICH management was estimated using Kaplan–Meier analyses. Statistical analyses were performed with R software, version 4.1.

## Results

During the study period, we included 460 patients with ICH, of whom 255 (55.4%) were male. The median age of the overall cohort was 79 years (IQR 69–85). The median NIHSS score at ICH onset was 10 (IQR 4–15), while the median Glasgow Coma Scale (GCS) score was 14 (IQR 8–15). Within 30 days, 134 patients (29.1%) died (Table 1).

**Table 1:** Main characteristics of the population

Males, n (%) [460]	255 (55.4)
Age, median (IQR) [460]	79 (69–85)
Hospitalization setting, n (%) [460]	
Neurology / Stroke Unit	219 (47.6)
Internal Medicine	62 (13.5)
Neurosurgery	68 (14.8)
Intensive Care Unit	60 (13.0)
Other	51 (11.1)
Risk factors, n (%)	
Arterial hypertension [457]	345 (75.5)
Dyslipidemia [450]	132 (29.3)
Diabetes mellitus [458]	95 (20.7)
Atrial fibrillation [455]	101 (22.2)
Obesity [438]	37 (8.4)
Cigarette smoking [449]	39 (8.7)
Alcohol abuse [444]	45 (10.1)
Ongoing treatments at onset, n (%)	
Lipid-lowering drugs [454]	111 (24.4)
Antihypertensives [460]	343 (74.6)
Antiplatelets [449]	181 (40.3)
Anticoagulants [449]	70 (15.6)
Pre-ICH mRS score, n (%) [341]	
0	197 (57.8)
1	63 (18.5)
2	21 (6.2)
3	18 (5.3)
4	18 (5.3)
5	24 (7.0)
NIHSS at ICH onset, median (IQR) [385]	10 (4–15)
GCS at ICH onset, median (IQR) [450]	14 (8–15)
Blood pressure on admission (mmHg), median (IQR)	
Systolic	156 (133–180)
Diastolic	90 (79–100)
Blood glucose on admission (mg/dL), median (IQR)	130 (103–169)
Body temperature on admission (°C), median (IQR)	36.3 (36.0–36.7)
30-day case-fatality, n (%) [460]	134 (29.1)

Numbers in square brackets identify the total number of available data

ICH, Intracerebral hemorrhage; IQR, Interquartile range; NIHSS, National Institutes of Health Stroke Scale; GCS, Glasgow Coma Scale

**Fig. 1:**
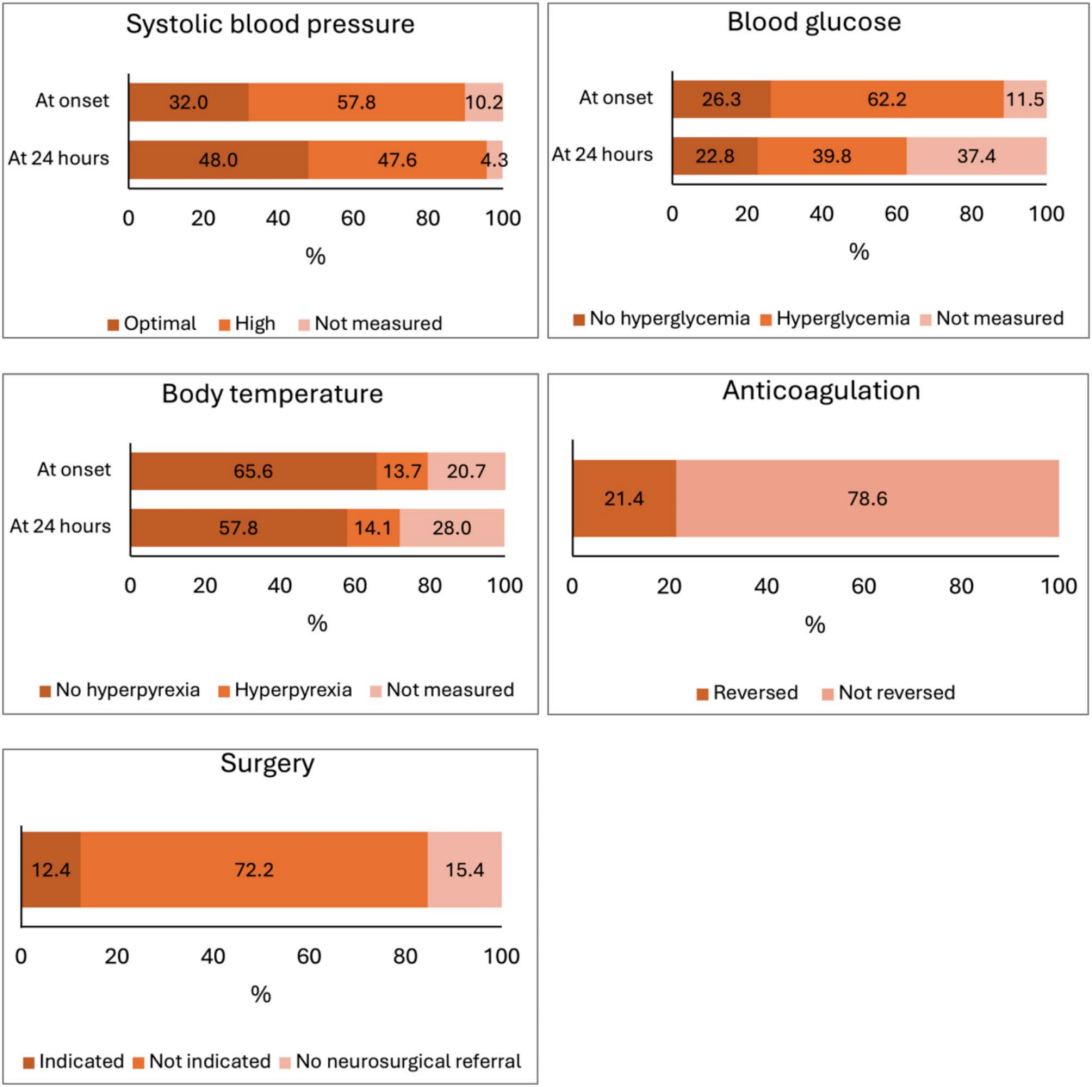
Parameters of intracerebral hemorrhage management during the first 24 h from symptom onset

SBP at onset was optimal in 147 patients (32.0%), high in 266 (57.8%), and not measured in 47 patients (10.2%). At ICH onset, 121 patients (26.3%) had no hyperglycemia, 286 (62.2%) had hyperglycemia, while blood glucose was not measured in 53 patients (11.5%). At ICH onset, 302 patients (65.6%) had no hyperpyrexia, 63 (13.7%) had hyperpyrexia, while body temperature was not measured in 95 patients (20.7%). At 24 h from ICH onset, 219 patients (47.6%) still had high SBP, 183 (39.8%) hyperglycemia, and 65 (14.1%) hyperpyrexia; SBP was not measured in 20 (4.3%) patients, blood glucose was not measured in 172 (37.4%) patients, and body temperature was not measured in 129 (28.0%) patients (Fig. 1).

Out of the 70 patients who were on anticoagulant treatment at ICH onset (41 with direct oral anticoagulants and 28 with vitamin K antagonists), 15 (21.4%) received anticoagulant reversal within 24 h from ICH onset.

Neurosurgical referral was asked for 389 (84.6%) patients, and 57 patients (12.4%) underwent hematoma evacuation or external ventricular drainage to treat hydrocephalus (Fig. 1).

**Fig. 2:**
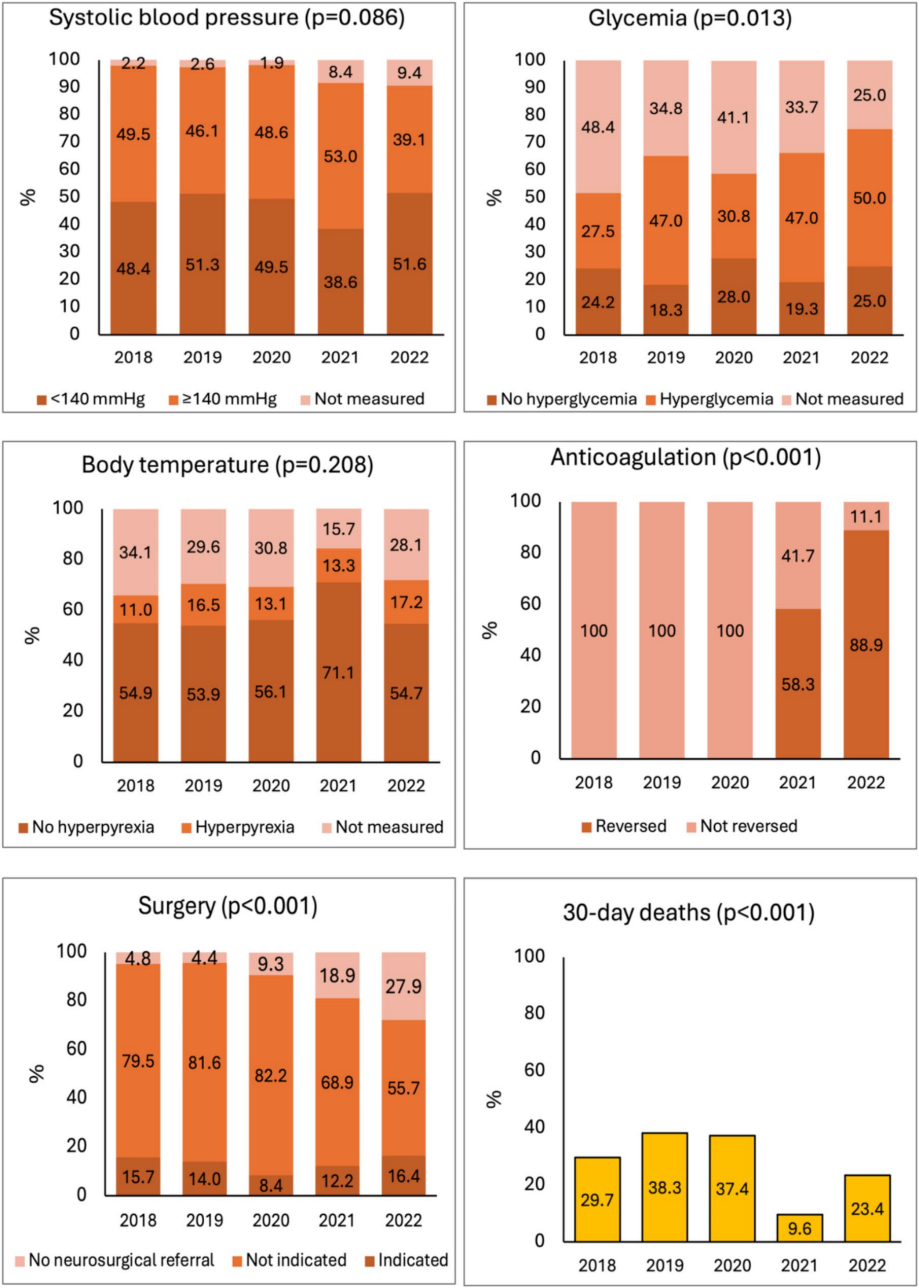
Trend in the control of parameters within 24 h from intracerebral hemorrhage onset over the study period

Figure 2 shows yearly trends in key parameters of ICH management at 24 h from ICH onset. We observed a change in the management of blood glucose (*p* = 0.013) driven by a decrease in the proportion of patients with blood glucose not measured, while the proportion of patients with no hyperglycemia did not change substantially. Anticoagulant reversal significantly increased over time (*p* < 0.001). Neurosurgical referrals changed over time (*p* < 0.001) driven by a decrease in neurosurgical referrals not followed by surgery, while the proportion of patients treated surgically remained stable. Thirty-day case-fatality rates showed a significant trend towards a decrease especially in 2021 and 2022 (*p* < 0.001).

**Fig. 3:**
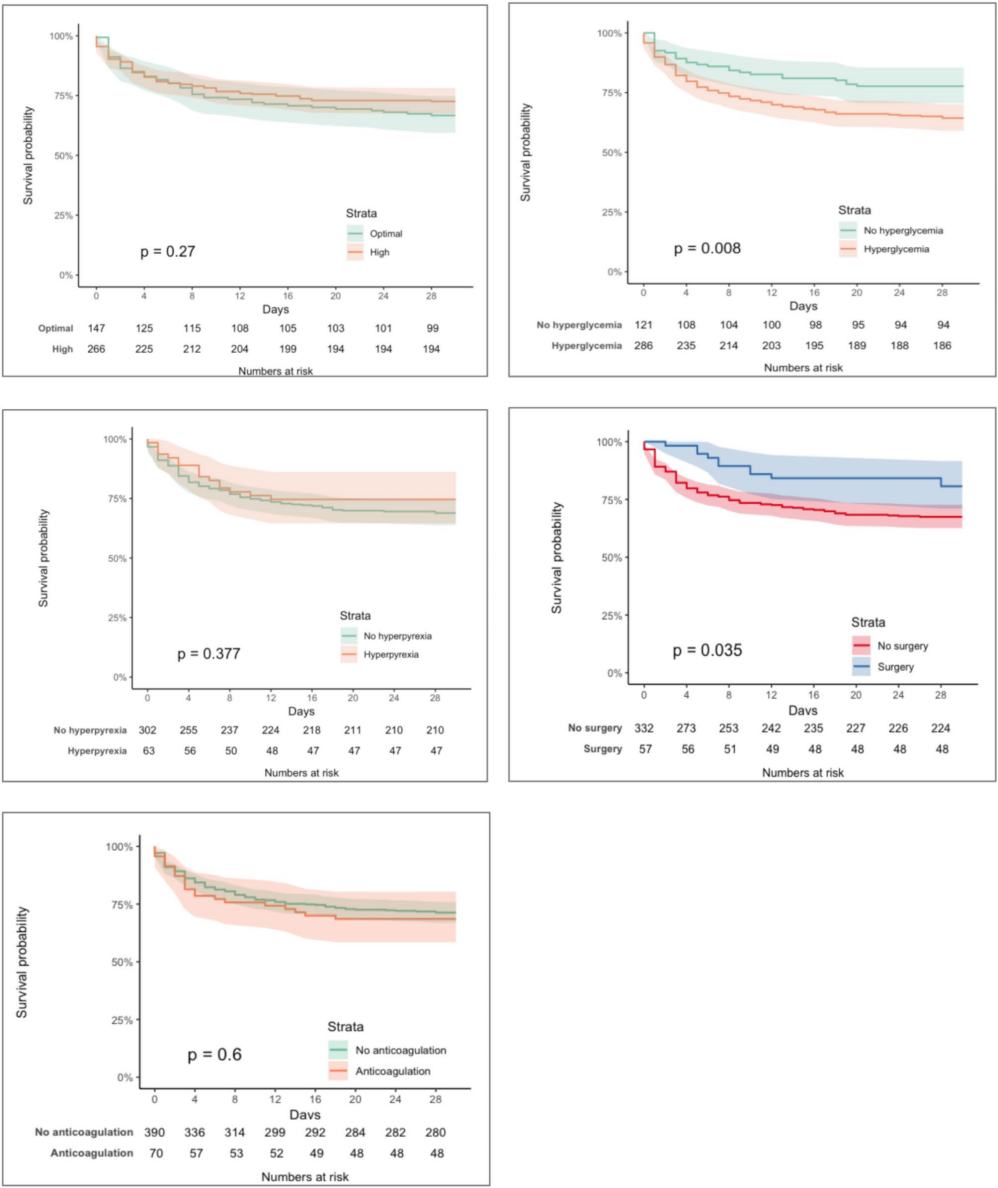
Kaplan-Meier curves of survival according to parameters of intracerebral hemorrhage management

At 30 days post-ICH, 72 patients with high SBP at onset (27.3%, 95% CI 21.3–34.3) and 50 patients with optimal SBP (33.6%, 95% CI 24.9–44.2) had died (p for Kaplan-Meier estimate = 0.270). Among patients with hyperglycemia, 102 (35.7%, 95% CI 29.1–43.3) died, compared to 27 patients without hyperglycemia (22.3%, 95% CI 14.7–32.5; *p* = 0.008). Additionally, 16 patients with hyperpyrexia (25.4%, 95% CI 14.5–41.2) died, compared to 94 patients without hyperpyrexia (31.1%, 95% CI 25.2–38.1; *p* = 0.377). Among patients treated with anticoagulants, 22 (31.4%, 95% CI 19.7–47.6) died, while 112 patients (28.7%, 95% CI 23.6–34.6) not treated with anticoagulants died (*p* = 0.600). Lastly, 11 patients who were treated surgically (19.3%, 95% CI 9.6–34.5) died, compared to 108 who were not treated (32.5%, 95% CI 26.7–39.3; *p* = 0.035). Figure 3 shows Kaplan-Meier estimates for each parameter of acute ICH management.

The characteristics and outcome did not differ between patients with SBP measured and not measured at ICH onset; patients with glycemia measured at ICH onset had higher 30-day case-fatality and a different distribution of hospitalization settings compared with those without glycemia measured at ICH onset; patients with body temperature measured at ICH onset had a higher prevalence of cigarette smoking at ICH onset compared with those without body temperature measured at ICH onset (Supplementary Table 1).

## Discussion

Our study documented a suboptimal compliance to acute ICH therapeutic measures in an Italian real-world population of patients with ICH over 5 years even if showing some trend towards improvements over the study period. Suboptimal compliance was due not only to the lack of control of some parameters, but also to the lack of measurement of some of them. Changes in ICH management could be due to better awareness among clinicians on therapeutic strategies that emerged during the study period from relevant clinical studies on ICH bundles of care. In the same period, new antidotes for direct oral anticoagulants were approved, leading to better management of ICH in the acute phase. However, during the same period, international guidelines and local management protocols for ICH did not change.

The timeframe of the present study (2018–2022) coincided with a transition in ICH care. The publication in 2019 of the first results on a bundle of care approach [4] encouraged awareness and improved practices of acute ICH management. Those improvements might have influenced a gradual improvement in ICH acute management, even in our population, despite organizational challenges.

A crucial point to interpret our findings is the setting of our study. Our population is served by a public healthcare system which guarantees equal access to care to the whole population. Therefore, our population is comparable to those of other public healthcare systems, while comparability to private healthcare systems is limited. The availability of Emergency Medical Services and public hospitals is widespread over the district; therefore, the spread of new therapeutic measures such as anticoagulant reversal rapidly reached the entire population. The population is served by one neurosurgical ward that serves as referral for the whole regionwith potential logistic consequences on referrals. Of note, the district is a mountainous area in which transportations might be difficult in some cases.

Several gaps from ideal ICH therapeutic targets [2] were identified in our population. Firstly, two-thirds of the ICH patients in our study presented with high SBP at onset, yet less than half achieved optimal SBP within the first 24 h. The INTERACT3 trial emphasized the importance of early and aggressive SBP reduction after ICH onset to significantly improve functional outcomes [7]. The challenges we observed in our real-world setting, such as delays in treatment and organizational issues, may explain the suboptimal results, contrasting with the more controlled environment of randomized trials.

Another notable finding was the low rate of anticoagulation reversal within 24 h of ICH onset and limited neurosurgical interventions. In patients on anticoagulation therapy, timely reversal is critical to preventing hematoma expansion and improving outcomes [13]. However, our study found that only 21.4% of anticoagulated patients received anticoagulation reversal within 24 h, potentially contributing to the high case-fatality rate. Other real-world studies have reported similar findings, with up to 40% of patients not receiving timely reversal, particularly in non-tertiary care centers [14]. This could be due to several factors, such as the availability of specific reversal agents (e.g., andexanet alfa, idarucizumab), or the complexity of decision-making based on ICH characteristics and patients’ comorbidities.

Neurosurgical interventions are essential in managing ICH, particularly in cases involving mass effect, elevated intracranial pressure, or obstructive hydrocephalus [15]. The Early Minimally Invasive Removal of Intracerebral Hemorrhage trial (ENRICH) demonstrated that early minimally invasive hematoma evacuation can improve functional outcomes [16]. However, our study revealed a gap between neurosurgical referrals and the actual number of surgeries performed, suggesting that many referrals may have been unnecessary, while others who could have benefited from surgery did not receive it. Establishing clear criteria for neurosurgical referrals is critical for optimizing patient outcomes and reducing unnecessary strain on neurosurgical services [2, 17].

Hyperglycemia is also a common occurrence in the acute phase of ICH and is linked to worse outcomes, such as increased mortality and poor functional recovery, which aligns with our findings [18–20]. The INTERACT3 trial included glycemic control as part of its bundle of care, although it did not isolate the effect of glycemic control alone [7]. Managing hyperglycemia in ICH is challenging due to the prevalence of stress-induced hyperglycemia, which complicates the distinction from chronic hyperglycemia and negatively affects timely interventions [12, 21–23]. In our study, 62.2% of patients had hyperglycemia at onset, but only 22.8% achieved glucose control within 24 h, highlighting the need for a better approach to managing hyperglycemia in the acute phase of ICH.

Another critical factor in ICH management is controlling hyperpyrexia, whether from hyperthermia or infection-related fever. Proper temperature management has been shown to improve outcomes by reducing secondary brain injury and limiting metabolic demands [24, 25]. In our study, 13.7% of patients presented with hyperpyrexia, rising to 14.1% within 24 h. While not originally part of early care bundles [4], recent trials like INTERACT3 have recognized its importance and incorporated it into the bundle of care, reinforcing the need for effective temperature management to improve outcomes in ICH patients [7].

Our time trends analysis over a 5-year period disclosed SBP and temperature control remained relatively stable, improvements in glycemic measurement, and more selective neurosurgical consultations, leading to better use of neurosurgical resources. We did not observe clear patterns of differences between patients with and without measured parameters at ICH onset.

Additionally, there was an increase in the use of anticoagulation reversal agents, likely driven by better awareness on the potential benefits of this strategy and on the availability of specific antidotes for direct oral anticoagulants. Importantly, this period saw a decrease in 30-day case-fatality, particularly in the years 2021–2022, which coincided with an increase in anticoagulation reversal. These findings suggest that improvements in acute ICH management can lead to better outcomes and reduced mortality.

Our study has several limitations. First, we did not assess therapeutic measures beyond the initial 24 h. Second, we measured key parameters at only two time points, limiting our ability to capture fluctuations within the first hours after onset. Third, the exact time from symptom onset to parameter measurement was not recorded, introducing variability in our data. Fourth, as this was a purely observational study, certain parameters (e.g., blood glucose, body temperature) were not consistently measured across all patients. We were unable to account for the specific reasons behind these omissions, as such decisions were made at the discretion of the treating physicians in the context of usual clinical practice. Fifth, we did not track the specific medications used to control blood pressure or blood glucose, which may have influenced outcomes. Lastly, we collected data solely on 30-day case-fatality, while post-ICH functional status – an essential outcome for evaluating ICH prognosis – was not recorded.

## Conclusions

Our study showed that there are opportunities to improve ICH management in real-world settings where bundles of care for ICH have not been implemented. Critical parameters such as blood pressure, blood glucose, and body temperature were not adequately controlled in a substantial proportion of patients within the first 24 h of ICH onset, which likely contributed to high case-fatality rates. These findings emphasize the need for structured interventions to improve the timely and effective management of these key factors. Additionally, the low rates of anticoagulation reversal and neurosurgical interventions reveal significant gaps in current clinical practice, indicating a need for better organizational processes and improved access to essential treatments. Addressing these challenges is crucial to enhancing patient’ outcomes and reducing ICH-related mortality.

## Electronic Supplementary Material

Below is the link to the electronic supplementary material.

Supplementary Material 1

## Data Availability

The dataset analysed during the current study are available from the corresponding author on reasonable request.
