# Supplementary material for: How far are we from bringing intensive care bundle for intracerebral hemorrhage into the real-world setting? A 5-year population based-study
Source: Neurol Sci. 2025 Mar 31;46(7):3147–55. doi: 10.1007/s10072-025-08113-x (PMC12152059; doi:10.1007/s10072-025-08113-x)
Supplement: Supplementary file 1 — Supplementary Material 1 [file 10072_2025_8113_MOESM1_ESM.docx]

Supplementary Table 1. Comparisons between patients with parameters checked and not checked at baseline.

| **Characteristic / Outcome** | **Systolic blood pressure measured**  **(n=413)** | **Systolic blood pressure not measured**  **(n=47)** | **p value** |
| --- | --- | --- | --- |
| Males, n (%) [460] | 226 (54.7) | 29 (61.7) | 0.449 |
| Age, median (IQR) [460] | 79 (69-85) | 74 (65-83) | 0.117 |
| Hospitalization setting, n (%) [460] |  |  | 0.850 |
| Neurology / Stroke Unit | 200 (48.4) | 19 (40.4) |  |
| Internal Medicine | 54 (13.1) | 8 (17.0) |  |
| Neurosurgery | 60 (14.5) | 8 (17.0) |  |
| Intensive Care Unit | 54 (13.1) | 6 (12.8) |  |
| Other | 45 (10.9) | 6 (12.8) |  |
| Risk factors, n (%) |  |  |  |
| Arterial hypertension [457] | 312 (76.1) | 33 (70.2) | 0.478 |
| Dyslipidemia [450] | 116 (28.8) | 16 (34.0) | 0.562 |
| Diabetes mellitus [458] | 88 (21.4) | 7 (14.9) | 0.393 |
| Atrial fibrillation [455] | 92 (22.5) | 9 (19.6) | 0.790 |
| Obesity [438] | 30 (7.6) | 7 (15.9) | 0.112 |
| Cigarette smoking [449] | 34 (8.4) | 5 (11.1) | 0.741 |
| Alcohol abuse [444] | 14 (3.6) | 4 (9.1) | 0.183 |
| Pre-ICH mRS score, n (%) [341] |  |  | 0.767 |
| 0 | 177 (57.3) | 20 (62.5) |  |
| 1 | 56 (18.1) | 7 (21.9) |  |
| 2 | 19 (6.1) | 2 (6.2) |  |
| 3 | 18 (5.8) | - |  |
| 4 | 17 (5.5( | 1 (3.1) |  |
| 5 | 22 (7.1) | 2 (6.2) |  |
| NIHSS at ICH onset, median (IQR) [385] | 10 (4-15) | 6 (3-11) | 0.080 |
| GCS at ICH onset, median (IQR) [450] | 14 (8-15) | 14 (11-15) | 0.301 |
| Blood pressure on admission (mmHg), median (IQR) |  |  |  |
| Systolic | 160 (135-180) | - | - |
| Diastolic | 90 (75-100) | - | - |
| Blood glucose on admission (mg/dL), median (IQR) | 131 (105-169) | 125 (93-159) | 0.137 |
| Body temperature on admission (°C), median (IQR) | 36.3 (36-36.7) | 36.5 (36.1-36.8) | 0.133 |
| 30-day case-fatality, n (%) [460] | 122 (29.5) | 12 (25.5) | 0.686 |

| **Characteristic / Outcome** | **Glycemia measured**  **(n=407)** | **Glycemia not measured**  **(n=53)** | **p value** |
| --- | --- | --- | --- |
| Males, n (%) [460] | 232 (57.0) | 23 (44.4) | 0.084 |
| Age, median (IQR) [460] | 79 (69-85) | 76 (68-82) | 0.198 |
| Hospitalization setting, n (%) [460] |  |  | 0.047 |
| Neurology / Stroke Unit | 186 (45.7) | 33 (62.3) |  |
| Internal Medicine | 61 (15.0) | 1 (1.9) |  |
| Neurosurgery | 61 (15.0) | 7 (13.2) |  |
| Intensive Care Unit | 52 (12.8) | 8 (15.1) |  |
| Other | 47 (11.5) | 4 (7.5) |  |
| Risk factors, n (%) |  |  |  |
| Arterial hypertension [457] | 303 (75.0) | 42 (79.2) | 0.613 |
| Dyslipidemia [450] | 121 (30.5) | 11 (20.8) | 0.194 |
| Diabetes mellitus [458] | 85 (21.0) | 10 (18.9) | 0.859 |
| Atrial fibrillation [455] | 90 (22.4) | 11 (20.8) | 0.926 |
| Obesity [438] | 33 (8.6) | 4 (7.5) | >0.999 |
| Cigarette smoking [449] | 35 (8.8) | 4 (7.5) | 0.957 |
| Alcohol abuse [444] | 18 (4.7) | - | 0.218 |
| Pre-ICH mRS score, n (%) [341] |  |  | 0.142 |
| 0 | 169 (58.5) | 28 (53.8) |  |
| 1 | 47 (16.3) | 16 (30.8) |  |
| 2 | 19 (6.6) | 2 (3.8) |  |
| 3 | 15 (5.2) | 3 (5.8) |  |
| 4 | 16 (5.5) | 2 (3.8) |  |
| 5 | 23 (8.0) | 1 (1.9) |  |
| NIHSS at ICH onset, median (IQR) [385] | 9 (4-15) | 12 (3-20) | 0.330 |
| GCS at ICH onset, median (IQR) [450] | 14 (8-15) | 15 (10-15) | 0.096 |
| Blood pressure on admission (mmHg), median (IQR) |  |  |  |
| Systolic | 160 (134-180) | 160 (135-180) | 0.983 |
| Diastolic | 90 (75-100) | 90 (80-100) | 0.333 |
| Blood glucose on admission (mg/dL), median (IQR) | 130 (103-169) | - | - |
| Body temperature on admission (°C), median (IQR) | 36.4 (36-36.7) | 36.0 (36.0-36.8) | 0.308 |
| 30-day case-fatality, n (%) [460] | 129 (31.7) | 5 (9.4) | 0.001 |

| **Characteristic / Outcome** | **Body temperature measured**  **(n=365)** | **Body temperature not measured**  **(n=95)** | **p value** |
| --- | --- | --- | --- |
| Males, n (%) [460] | 208 (57.0) | 47 (49.5) | 0.232 |
| Age, median (IQR) [460] | 79 (69-85) | 77 (70-85) | 0.407 |
| Hospitalization setting, n (%) [460] |  |  | 0.066 |
| Neurology / Stroke Unit | 168 (46.0) | 51 (53.7) |  |
| Internal Medicine | 53 (14.5) | 9 (9.5) |  |
| Neurosurgery | 59 (16.2) | 9 (9.5) |  |
| Intensive Care Unit | 50 (13.7) | 10 (10.5) |  |
| Other | 35 (9.6) | 16 (16.8) |  |
| Risk factors, n (%) |  |  |  |
| Arterial hypertension [457] | 274 (75.7) | 71 (74.7) | 0.953 |
| Dyslipidemia [450] | 110 (31.0) | 22 (23.2) | 0.173 |
| Diabetes mellitus [458] | 76 (20.9) | 19 (20.0) | 0.953 |
| Atrial fibrillation [455] | 80 (22.2) | 21 (22.3) | >0.999 |
| Obesity [438] | 27 (7.8) | 10 (10.9) | 0.466 |
| Cigarette smoking [449] | 37 (10.3) | 2 (2.2) | 0.024 |
| Alcohol abuse [444] | 18 (5.2) | - | 0.061 |
| Pre-ICH mRS score, n (%) [341] |  |  | 0.394 |
| 0 | 144 (55.4) | 53 (65.4) |  |
| 1 | 51 (19.6) | 12 (14.8) |  |
| 2 | 16 (6.2) | 5 (6.2) |  |
| 3 | 14 (5.4) | 4 (4.9) |  |
| 4 | 13 (5.0) | 5 (6.2) |  |
| 5 | 22 (8.5) | 2 (2.5) |  |
| NIHSS at ICH onset, median (IQR) [385] | 10 (4-15) | 10 (3-14) | 0.576 |
| GCS at ICH onset, median (IQR) [450] | 14 (8-15) | 14 (10-15) | 0.123 |
| Blood pressure on admission (mmHg), median (IQR) |  |  |  |
| Systolic | 160 (135-180) | 155 (133-185) | 0.872 |
| Diastolic | 90 (75-100) | 90 (75-100) | 0.654 |
| Blood glucose on admission (mg/dL), median (IQR) | 131 (105-170) | 126 (99-158) | 0.329 |
| Body temperature on admission (°C), median (IQR) | 36.3 (36.0-36.7) | - | - |
| 30-day case-fatality, n (%) [460] | 110 (30.1) | 24 (25.3) | 0.421 |
